# Quantifying Vulnerability to Extreme Heat in Time Series Analyses: A Novel Approach Applied to Neighborhood Social Disparities under Climate Change

**DOI:** 10.3390/ijerph120911869

**Published:** 2015-09-22

**Authors:** Tarik Benmarhnia, Patrick Grenier, Allan Brand, Michel Fournier, Séverine Deguen, Audrey Smargiassi

**Affiliations:** 1 Université de Montréal, DSEST, Montréal, QC H3T 1A8, Canada; E-Mail: audrey.smargiassi@umontreal.ca; 2 EHESP School of Public Health, Rennes, Sorbonne-Paris Cité 35043, France; E-Mail: severine.deguen@ehesp.fr; 3 Institute for Health and Social Policy, McGill University, Montreal QC H3A 1A3, Canada; 4 Consortium Ouranos, Montréal, Département Scénarios et Services Climatiques, Montréal, QC H3A 1B9, Canada; E-Mail: grenier.patrick@ouranos.ca; 5 Institut National de Santé Publique du Québec, Montréal, QC H3A 1B9, Canada; E-Mail: allan.brand83@gmail.com; 6 Direction de santé publique de l’Agence de la santé et des services sociaux de Montréal, QC H2P 1E2, Canada; E-Mail: mfournie@santepub-mtl.qc.ca; 7 INSERM U1085 (IRSET), Rennes 35042, France

**Keywords:** social health inequalities, years of life lost, climate change, climate models, population health, temperature, time-series analyses

## Abstract

*Objectives*: We propose a novel approach to examine vulnerability in the relationship between heat and years of life lost and apply to neighborhood social disparities in Montreal and Paris. *Methods*: We used historical data from the summers of 1990 through 2007 for Montreal and from 2004 through 2009 for Paris to estimate daily years of life lost social disparities (DYLLD), summarizing social inequalities across groups. We used Generalized Linear Models to separately estimate relative risks (RR) for DYLLD in association with daily mean temperatures in both cities. We used 30 climate scenarios of daily mean temperature to estimate future temperature distributions (2021–2050). We performed random effect meta-analyses to assess the impact of climate change by climate scenario for each city and compared the impact of climate change for the two cities using a meta-regression analysis. *Results*: We show that an increase in ambient temperature leads to an increase in social disparities in daily years of life lost. The impact of climate change on DYLLD attributable to temperature was of 2.06 (95% CI: 1.90, 2.25) in Montreal and 1.77 (95% CI: 1.61, 1.94) in Paris. The city explained a difference of 0.31 (95% CI: 0.14, 0.49) on the impact of climate change. *Conclusion*: We propose a new analytical approach for estimating vulnerability in the relationship between heat and health. Our results suggest that in Paris and Montreal, health disparities related to heat impacts exist today and will increase in the future.

## 1. Introduction

It is well known that rising ambient temperatures are associated with increased heat related health impacts, namely increases in mortality or years of life lost [1,2]. This is an imperative public health matter, especially in the context of climate change [3]. Yet, heat related mortality is not equally distributed across populations or territories within cities. Populations or territories that are more impacted by heat are considered as vulnerable, where vulnerability is related to a factor that modifies the effect of heat on health outcomes (e.g., mortality) [4]. One such factor is related to neighborhood social disparities measured through neighborhood level of education or income for example [5,6,7,8]. The evidence about this vulnerability factor is still not clear since certain studies found an effect modification, while others did not [9]. Neighborhood level of education or income can represent upstream cause of health disparities, since health disparities can be explained by other determinants of health. Yet, identifying social disparities related to such upstream determinants of health are still useful to design targeted interventions aiming at reducing such disparities.

Assessing effect modification in the relationship between heat and health in order to identify heat-vulnerability factors is essential in the development of specific policies included in heat action plans [10]. In addition, heat related mortality projections for vulnerable populations can be useful to orient the allocation of resources towards those who are most in need in order to reach health equity, and to orient the implementation of adaptation measures to reach social justice ideals in the context of climate change.

Times series studies are the most commonly used type of study when assessing the association between temperature and health outcomes [9,10,11,12,13]. A wide majority of time series studies investigating potential effect modifiers in the relation between heat and mortality used the stratification to highlight heterogeneity in this relation [9]. In addition, it is important to mention that many of these studies base their conclusions about vulnerability on finding a statistically significant association in one stratum but not the other [14], an approach that is statistically invalid.

Here, we propose a novel approach to obtain a single index to represent effect modification (*i.e.*, vulnerability) in the relationship between heat and years of life lost. The main idea of this approach is to address the association between temperature and the intra-population disparities directly, instead of assessing if the heat-mortality association differs across two or more strata. This approach would prevent the loss of statistical power, and provide a comprehensive interpretation about vulnerability to heat related mortality.

We labeled this index Daily Years of Life Lost Disparities (DYLLD). As a case study, we compared neighborhood SES (measured through neighborhood level of education) potential vulnerability between Montreal and Paris. We also used this estimate to assess such disparities projections under climate change using a large ensemble of climate scenarios.

## 2. Methods

### 2.1. Data Source

We included all residents of the island of Montreal, Canada who died in the city in the summers (June, July and August) of 1990 to 2007 and the residents of the city of Paris, France in the summers of 2004–2009. For Montreal, we included all underlying non-accidental causes of death. It was not possible to exclude accidental causes of death in Paris, because we did not have access to underlying causes of mortality. Therefore we only included Paris subjects older than 35 years old at the time of death to minimize this bias, because accidental causes of death are dominant in subjects younger than 35 years [15]. To calculate the life expectancy at birth for each death, we used the Quebec life table for Montreal for the years 2000 to 2002 [16] and the French life table for Paris for the years 2004 to 2006 [17], matching by age and sex. We then summed all the YLL individual estimates by day to obtain the total daily YLL.

We attributed the following neighborhood SES indicators to each death at the smallest census unit area available. For Montreal, we used the percentage of the population of a dissemination area aged >20 years without a high school diploma from the 2006 census [18], and for Paris, we used the percentage of the population without a high school diploma from the French census division (group of blocks for statistical information) from the 2006 national census [17]. Daily mean outdoor temperatures (°C) and daily relative humidity (%) were obtained for the period 1981–2010 from Environment Canada [19] for Montreal and from Météo-France [20] for Paris.

### 2.2. Daily Years of Life Lost Disparities (DYLLD) Computation

We first stratified the total daily YLL (as described above) by terciles of the indicator of neighborhood SES vulnerability. We then used a modified “Index of Disparity” [21], to summarize disparities across groups (here neighborhood SES groups). We calculated daily YLL disparities (DYLLD) with Formula (1):

(1) $$DYLLD=[\;\overset{J}{\underset{j=1}{\sum }}\left(\text{d}YLL_{j}-\text{ d}YLL_{ref}\right)\;/\;J\;]$$

where *dYLL* is the total daily years of life lost, *j* indexes each of the J neighborhood SES groups (in addition to the reference group, here *J* = 2), and *dYLL_ref_* is the daily total *dYLL* estimate for the reference group (here the highest neighborhood SES group). Using this index allowed us to obtain a single daily estimate of *dYLL* social disparities between the different neighborhood SES strata. This index thus represents a daily average difference between all groups and the most advantaged neighborhood SES group.

### 2.3. Estimating the Association between DYLLD and Temperature in Montreal and Paris

The relationship between DYLLD and daily mean temperatures in both cities separately was modeled with a non-parametric approach, with adjustments for trend, season, and daily humidity. Details about the statistical modeling are provided in supplementary material. We calculated Relative Risks (RR) for the relation between DYLLD and daily temperature relative to the average DYLLD for the entire period [5]. We estimated RRs by each temperature unit. We calculated rates (per 100,000 individuals) of DYLLD attributable to temperature for each city. Details about the calculation of DYLLD attributable to temperature are provided in supplementary material.

### 2.4. Using DYLLD in Projections under Climate Change

We defined the historical period as 1981–2010 and the future period as 2021–2050. Thirty climate scenarios of daily mean temperature were used (details in Supplementary Material: Table S1). A climate scenario is defined here as a time series with statistical properties judged plausible over a period of time. See Supplemental Material for further information about climate scenarios of historical and future temperatures. We defined the impact of climate change (ICC) on DYLLD attributable to temperature as the ratio between future and historical summer estimates. We then performed random effect meta-analyses of ICC by climate scenario to produce a pooled ICC (and its 95% CI) for each city separately (see Supplementary Material for the formula used to calculate the standard errors). We assumed no acclimatization to heat, and no changes in population size, age structure, neighborhood SES disparities or life expectancy. We first compared the impact of climate change on temperatures between the two cities. We conducted random effect meta-analyses of averaged difference between observed historical and future daily mean temperatures by climate scenario (*n* = 30). We computed pooled estimates for each city and compared them (using a *t*-test). We compared historical and future DYLLD rates (per 100,000 individuals) attributable to temperature between Montreal and Paris (see details in supplementary material). We then conducted a meta regression analysis on the 60 future rates (30 for Montreal and 30 for Paris) where the dependent variable was the ln (DYLLD rates) and the independent variable was the city. Different sensitivity analyses were conducted and are presented in supplementary Material.

## 3. Results

**Figure 1:**
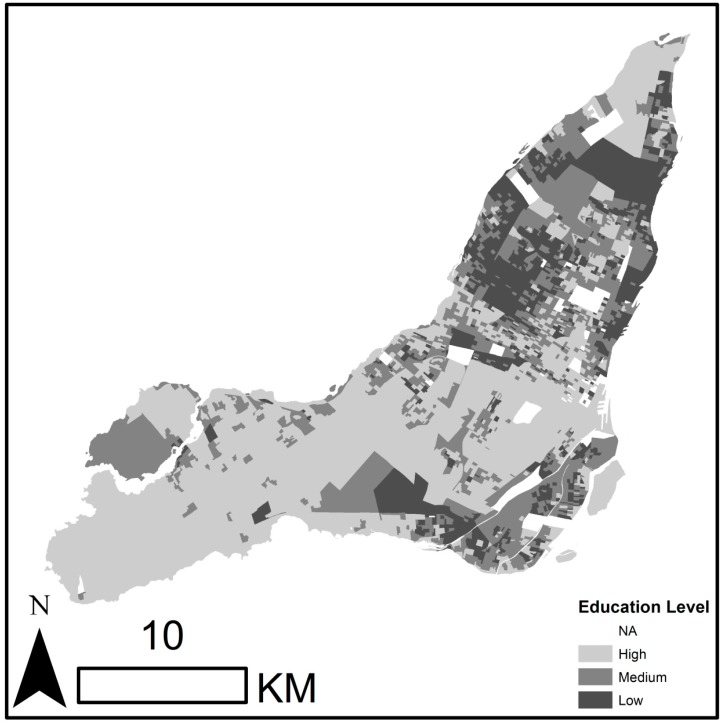
The spatial distribution of neighborhood social disparities in Montreal.

**Figure 2:**
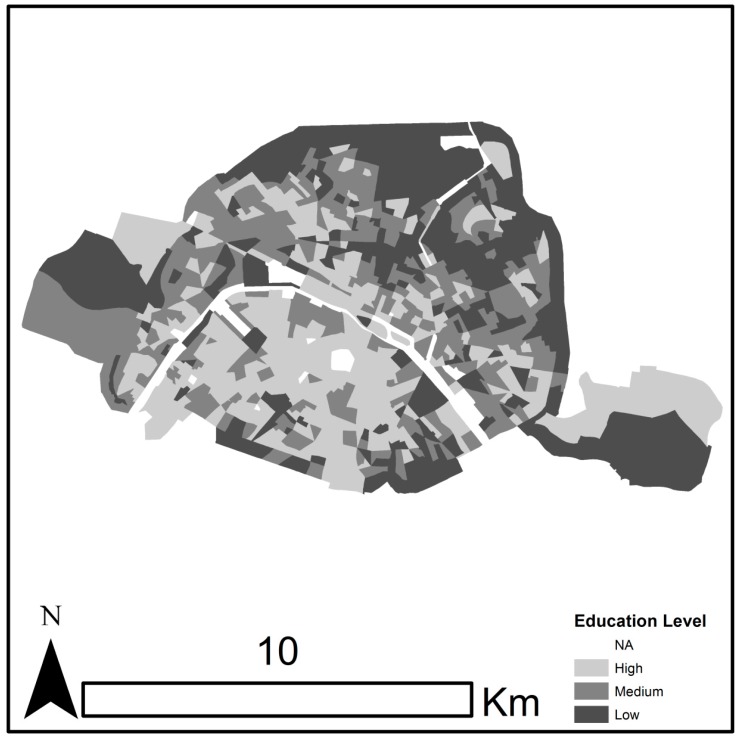
The spatial distribution of neighborhood social disparities in Paris.

Each year an average of 3408 and 3235 deaths (representing 188 and 145 deaths per 100 000 persons) during the summer months (June–August) occurred in Montreal and Paris, respectively, during the study periods (1990–2007 for Montreal and 2004–2009 for Paris). The average summer daily mean observed temperatures were 20.4 °C (SD = 3.24 °C) for Montreal and 19.63 °C (SD = 3.21 °C) for Paris for the study observation periods. The daily YLL ranged from 1076 years to 6739 years in Montreal (mean = 2660 years) and from 633 years to 2991 years in Paris (mean = 1483 years). Figure 1 and Figure 2 present the spatial distribution of neighborhood social disparities in Montreal and Paris respectively. The percentage of the population living in low neighborhood SES ranged from 0% to 73.47% in Montreal (mean = 20.97%) and from 0% to 100% in Paris (mean = 33.67%) (details in Supplementary Material: Table S2). Table S3 presents estimates of Daily Years of Life Lost Disparities (DYLLD) in Montreal and Paris. The mean daily estimates of DYLLD were 339.35 years (SD = 326.21 years) for Montreal and 187.32 years (SD = 169.11 years) for Paris. Descriptive statistics for daily mean temperatures from the 30 climate scenarios, respectively, in Montreal and Paris, for the periods 1981–2010 and 2021–2050 are presented in Supplementary Material (Tables S4 and S5).

The pooled estimate of the averaged difference between historical and future scenario daily mean temperatures was 1.35 °C (95% CI: 1.13 °C, 1.60 °C) for Montreal and 1.12 °C (95% CI: 0.93 °C, 1.36 °C) for Paris suggesting that daily mean temperature increase will be larger (*p* < 0.01) in Montreal than in Paris according to the climate scenarios included in our analysis.

The relations between daily temperatures and DYLLD in Paris and Montreal were J-shaped. The effect of temperature started to increase at 23 °C in Montreal and 22 °C in Paris (see Tables S6 and S7). We found no effect of temperatures on DYLLD below these respective thresholds. These estimates correspond to the effect measure modification of the neighborhood level education on the association between temperature and years of life lost.

**Figure 3:**
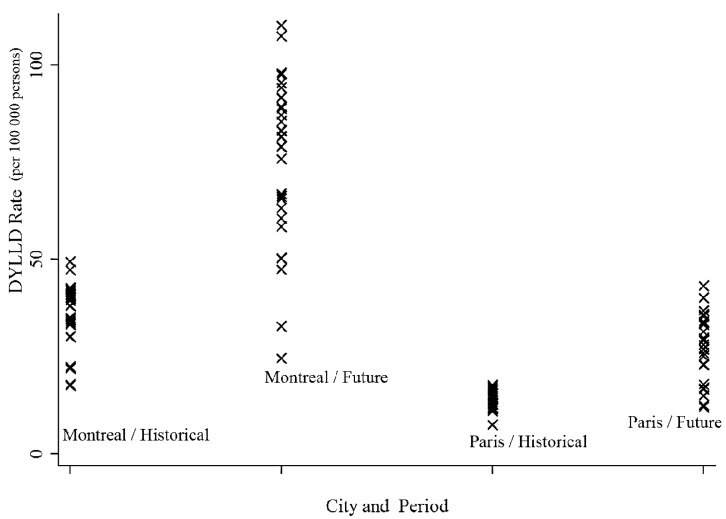
Historical and projected estimates of rates of Daily Years of Life Lost Disparities (DYLLD) attributable to temperature for Montreal and Paris.

The summer rate of DYLLD attributable to temperature in historical period was greater in Montreal (34.70 years per 100,000 persons; 95% CI: 16.22, 47.44) than in Paris (13.34 years per 100,000 persons; 95% CI: 7.97, 18.26) (*p* < 0.01). Figure 3 presents the historical and projected estimates of rates of DYLLD attributable to temperature for Montreal and Paris.

For the future period, rates of DYLLD attributable to future summer temperatures ranged from 22.40 years per 100,000 persons (95% CI: 14.01, 32.77) to 100.24 years per 100,000 persons (95% CI: 80.54, 121.75) in Montreal and from 9.98 years per 100,000 persons (95% CI: 6.00, 17.23) to 35.23 years per 100,000 persons (95% CI: 26.50, 41.18) in Paris. The future summer rate of DYLLD attributable to temperature was 45.13 years per 100,000 persons (95% CI: 37.17, 53.54) higher in Montreal than in Paris (Table 1).

**Table 1:** Meta-regression models investigating the influence of the city on future rates of summer DYLLD and on the impact of climate change.

	Influence of the City*	
Dependent Variable	Beta (95% CI)	*p* Value
Future summer rates of DYLLD** (2021-2050)	45.13*** (37.17 to 53.54)	**< 0.001**
Impact of Climate Change (ICC) on summer DYLLD	0.31 (0.14 to 0.49)	**< 0.001**

* Montreal was coded as 1 and Paris as 0; ** The total population used was 1,812,723 (year 2001) for Montreal and 2,234,105 (year 2006) for Paris; *** Summer rates of DYLLD are expressed per 100,000 persons; CI: Confidence Interval.

The ICC (*i.e.*, the ratio between future and historical DYLLD) was higher in Montreal 2.06 (95% CI: 1.90, 2.25) (Figure S1) than in Paris 1.77 (95% CI: 1.61, 1.94) (Figure S2). The ICC in Montreal and Paris differed by 0.31 (95% CI: 0.14, 0.49) (Table 1).

## 4. Discussion

In this paper, we showed that an increase in ambient temperature can lead to an increase in daily years of life lost disparities (according to the neighborhood SES) in both Montreal and Paris. We also report these heat-related neighborhood SES disparities to be larger in Montreal than in Paris in both historical (1981–2010) and future (2021–2050) periods. Lastly, we estimate that the increasing years of life lost neighborhood SES disparities associated with climate change will be higher in Montreal than in Paris. This last result can be partly explained by the fact that the impact of climate change on daily mean temperature will be larger in Montreal than in Paris according to the climate scenarios included in our analysis.

We propose a new approach to obtain a single index to represent effect modification in the relationship between heat and years of life lost. This index, which is based on the econometric literature [21] and modified for environmental epidemiology area, is simple to compute and can be used for a range of heat-related vulnerability factors and health outcomes. Further studies could use it to compare heat-related vulnerability across different contexts and to address the determinants of such vulnerabilities. This index represents a good alternative to stratified analyses, which are commonly used in this context [9,11]. Other methods such as the case-only approach [22] or the use of ratio of risk ratios (RRR) [9,23] could also be used to describe effect modification when assessing the effect of heat on health and for comparison across different contexts.

In parallel to the index we propose, other methods deserve mention. We calculated future health impacts, using a large panel of climate scenarios from the most recent IPCC report from global climate modeling centers with modern post-processing analyses (*i.e.*, quantile-quantile methods). Indeed, the use of numerous climate scenarios is essential to represent the uncertainty of future mortality impacts [24]. We also proposed a way to compare the heat-related health impacts of climate change in different cities by conducting meta-regressions.

In the case studies we present, we attempted to conduct equivalent analytical approaches between the two cities to keep the comparison plausible, yet there are several limitations to our comparative analysis. First, the urban configurations and social heritages are different between Montreal and Paris. Indeed, in the Montreal Metropolitan Area (Greater Montreal) which represents the reach of commuter movement to and from the island of Montreal and its surrounding suburbs, the most socially disadvantaged communities are situated on the island of Montreal [25], whereas in the Paris Metropolitan Area, the city of Paris (except a few neighborhoods in the city’s northeast) over represents the less socially disadvantaged communities of the Paris Metropolitan Area [26]. This could explain why we found more heat-related social disparities in Montreal than in Paris. Second, baseline age distributions and mortality rates are quite different between the two cities. These points could have an influence on the DYLLD attributable to historical and future temperatures comparison. However, they will most likely not affect our results on the impact of climate change on health disparities, because these estimates are relative to each city. Furthermore, using different sensitivity analyses about the mortality data and the social vulnerability factor used (see Table S8), our results remained unaltered. In addition, we did not consider population acclimatization to heat impacts in the observed period, which could overestimate our DYLLD estimates. Yet, such acclimatization over time is not likely to occur for Paris since the observed period was short (only between 2004 and 2009).

Other limitations of this study deserve mention and could be addressed in further studies. First, we did not consider the effect of heat waves [27], which can possibly result in our health impact projections being underestimated. Second, we only considered the heat effects on DYLLD at the current day’s temperature (*i.e.*, lag0), which may underestimate our results. However, according to previous studies, the strongest heat effects are from the current day’s temperature [28], thus this underestimation is likely to be small. We did not consider mortality displacement (or harvesting effect), which can possibly overestimate our results. Future studies could assess this point by using a partial fix [29] or displacement ratios techniques [30] for example. Third, we applied the historical associations between ambient temperature and DYLLD to future populations, and by doing this, we disregarded the repercussion of demographic or health changes (e.g., increase in life expectancy, changes in age structure) and adaptation measures which could influence the population’s vulnerability [31]. Yet, by conducting sensitivity accounting for different demographic projections, the conclusions of the study were not altered (see Figures S3–S6).

## 5. Conclusions

In this paper, we present a new index that can be used in different settings in time series analyses and will help to achieve future systematic epidemiologic evaluations about heat-related vulnerability and its determinants [32]. This index is applied to time series designs. Therefore, we strongly encourage further studies to develop analogous indexes for different study designs to facilitate the investigation of effect modification in epidemiology. Our index can be used with further potential vulnerability factors such as neighborhood level of poverty or green spaces cover. This index can also be used in many epidemiologic contexts where time series designs are commonly used such as studies of air pollution [33], or policy evaluation [34]. The development of methods that facilitate the investigation of effect modification is essential for the success of public health programs and their implementation for achieving health equity, and methodological improvements in such practices are still needed.

## Supplementary Files

Supplementary File 1
