# Supplementary material for: Quantifying Vulnerability to Extreme Heat in Time Series Analyses: A Novel Approach Applied to Neighborhood Social Disparities under Climate Change
Source: Int J Environ Res Public Health. 2015 Sep 22;12(9):11869–79. doi: 10.3390/ijerph120911869 (PMC4586712; doi:10.3390/ijerph120911869)
Supplement: Supplementary File 1 [file ijerph-12-11869-s001.pdf]

Supplementary Information

Quantifying Vulnerability to Extreme Heat in Time Series Analyses: A Novel Approach Applied to Neighborhood Social Disparities under Climate Change

---

Supplementary Methods

Climate Scenarios of Historical and Future Temperatures

Each climate scenario is based on a climate simulation from a numerical Earth System Model (ESM) used in the Coupled Model Intercomparison Project Phase 5 (CMIP5) [1]. The post-processing of each model's raw simulation is performed for increasing statistical agreement of temperature distributions with observations during the historical period, following a method akin to that of Themeßl *et al*. [2]. The same 30 climate simulations are used for Montreal and Paris, but post-processing steps are conducted separately, using each city's meteorological data. No single scenario should be seen as a prediction, but as an ensemble they aim to cover the real future climate trajectory. The selection of simulations covers 4 different emission scenarios and 7 different ESMs (see the list in Table S1).

Estimating the Historical Association between DYLLD and Temperature

A regression model was used to estimate the association between DYLLD and daily mean temperatures in both cities separately. We adjusted for seasonal effects and long-term trends using a natural cubic spline for time with 7 degrees of freedom (df) per year [3] in the DYLLD series. To consider the non-linear relationship between DYLLD and temperature, we modeled the temperature variable as a cubic spline with 3 knots (corresponding to 25th, 50th and 75th percentiles). Models were adjusted or not (for sensitivity analyses), for daily relative humidity. Sensitivity analyses were carried out by changing the degrees of freedom for secular trends. The adequacy of the models was checked by verifying that the residuals were independent over time (with visual inspection of partial autocorrelation plots and using white noise statistical test).

Calculation of DYLLD Attributable to Temperature

Attributable DYLLD were calculated using attributable fractions (AF) with the specific RRs by temperature, calculated with the historical data. We included in the calculation only days with daily temperature related to a RR > 1. To calculate the total DYLLD attributable to temperature we used the Formula (2):

$$\text{Total DYLLD}_{att} = \Sigma\ (\text{AF}(T_i) \times \text{Mean}_{\text{DYLLD}} \times \text{ND}(T_i)) \quad (2)$$

where $\text{Mean}_{\text{DYLLD}}$ is the mean observed DYLLD for the observed periods and $\text{ND}(T_i)$ is the number of days with the value of the temperature (observed or simulated) unit = T_i, and values are summed from the minimum value of T_i (for which the estimated RR was >1) to the maximum value of T_i.

Table S1. Climate model, emission scenario and member code for each simulation used in this study.

Simulation ID	Climate Model	Emission Scenarios	Member
1	BCC-CSM1.1	rcp26	r1i1p1
2	CanESM2	rcp26	r1i1p
3	CanESM2	rcp26	r2i1p1
4	CanESM2	rcp26	r3i1p1
5	CanESM2	rcp26	r4i1p1
6	CanESM2	rcp26	r5i1p1
7	MPI-ESM-LR	rcp26	r1i1p1
8	NorESM1-M	rcp26	r1i1p1
9	BCC-CSM1.1	rcp45	r1i1p1
10	CanESM2	rcp45	r1i1p1
11	CanESM2	rcp45	r2i1p1
12	CanESM2	rcp45	r3i1p1
13	CanESM2	rcp45	r4i1p1
14	CanESM2	rcp45	r5i1p1
15	INM-CM4	rcp45	r1i1p1
16	IPSL-CM5A-LR	rcp45	r1i1p1
17	MPI-ESM-LR	rcp45	r1i1p1
18	MRI-CGCM3	rcp45	r1i1p1
19	NorESM1-M	rcp45	r1i1p1
20	BCC-CSM1.1	rcp60	r1i1p1
21	IPSL-CM5A-LR	rcp60	r1i1p1
22	NorESM1-M	rcp60	r1i1p1
23	BCC-CSM1.1	rcp85	r1i1p1
24	CanESM2	rcp85	r1i1p1
25	CanESM2	rcp85	r2i1p1
26	CanESM2	rcp85	r3i1p1
27	INM-CM4	rcp85	r1i1p1
28	MPI-ESM-LR	rcp85	r1i1p1
29	MRI-CGCM3	rcp85	r1i1p1
30	NorESM1-M	rcp85	r1i1p1

We used the observed temperature distribution for the period 1981-2010 to estimate historical total DYLLD attributable to temperatures in Paris and in Montreal ($n = 1 \times 2$). We used simulated temperature distributions in both cities ($n = 30 \times 2$) for the period 2021-2050 to estimate future total DYLLD attributable to temperatures. We chose to present annual June–August estimates (referred to as summer estimates) by dividing the total DYLLD attributable to temperature by 30 (years) for both the historical and future periods. We used bootstrapping to construct the 95% confidence intervals for summer attributable number of DYLLD. We estimated percentile bootstrap 95% confidence intervals for the total attributable number of DYLLD with one thousand bootstrap samples based on the observed data, from which we selected the 2.5% and the 97.5% of the number of total DYLLD. We created bootstrap samples by choosing DYLLD randomly in each year among the whole periods.

Comparison of Historical and Future DYLLD Attributable to Simulated Temperature between Montreal and Paris

To compare historical summer DYLLD attributable to observed historical temperature between Montreal and Paris, we first calculated rates of DYLLD attributable to temperature, by dividing the total number of DYLLD attributable to temperature by the total population in each city on year 2001 for Montreal and year 2006 in Paris, based on census data [4,5]. We compared the two estimates by conducting a *t* test.

We also compared future summer DYLLD attributable to simulated temperature between Montreal and Paris. As for historical estimates, we calculated rates using the 2001 and 2006 populations.

Formula used to calculate the standard errors of the ratios

$$SD(ratio) = ratio \times \sqrt{\left(\frac{SD\ Future^2}{Future}\right) + \left(\frac{SD\ Historical^2}{Historical}\right)}$$

where SD is Standard Deviation; Future represents the YLL disparities attributable to temperature for the future period (2021–2050) and Historical represents the YLL disparities attributable to temperature for the historical period (1981–2010).

Supplementary Results

Table S2. Summary statistics for to neighbourhood education level in Montreal and Paris.

Variable	Mean	Minimum	25th Percentile	Median	75th Percentile	Maximum	Standard Deviation
Education Level (%) in Montreal	20.97	0	13.27	20.21	27.62	73.47	10.26
Education Level (%) in Paris	33.67	0	26.94	36.29	42.10	100	11.59

Table S3. Descriptive statistics of DYLLD in Montreal (1990–2007) and Paris (2004–2009).

	Mean	Minimum	Maximum	Std. Dev
DYLLD in Montreal	339.35	−527.41	1649.34	326.21
DYLLD in Paris	187.32	−300.56	829.25	169.11

Table S4. Descriptive statistics of daily mean temperatures from the 30 simulations in Montreal, for the periods 1981–2010 and 2021–2050, corrected with the quantile mapping method.

	**Montreal (1981–2010)**				**Montreal (2021–2050)**			
**Simulation ID**	**Mean**	**Minimum**	**Maximum**	**Std. Dev**	**Mean**	**Minimum**	**Maximum**	**Std. Dev**
1	20.07	8	31	3.60	21.20	7	34	3.45
2	20.49	9	31	3.44	21.72	10	33	3.31
3	19.97	9	30	3.28	21.81	9	32	3.28
4	19.94	8	32	3.33	22.02	10	33	3.40
5	19.98	9	30	3.42	21.94	10	32	3.45
6	20.08	6	30	3.40	21.72	10	33	3.50
7	20.07	8	29	3.49	21.30	10	33	3.79
8	20.10	9	29	2.86	21.05	10	30	2.91
9	20.08	8	32	3.56	20.85	9	34	3.60
10	20.26	9	31	3.36	21.79	10	33	3.41
11	20.06	8	33	3.36	22.01	9	34	3.58
12	19.99	8	29	3.30	21.77	11	35	3.46
13	20.08	9	30	3.41	21.91	10	32	3.37
14	20.10	6	31	3.41	21.81	7	36	3.63
15	20.10	11	28	2.63	20.40	11	31	2.74
16	20.14	8	28	3.10	22.01	11	29	3.16
17	20.11	8	29	3.45	21.58	9	32	3.52
18	20.15	9	31	3.20	20.94	10	33	3.11
19	20.12	10	29	2.82	21.61	11	30	2.88
20	20.07	8	31	3.59	21.17	9	33	3.48
21	20.15	8	28	3.08	21.66	6	30	3.21
22	20.12	10	29	2.84	21.15	10	30	2.94
23	20.08	9	31	3.61	21.52	9	36	3.69
24	20.38	9	31	3.44	22.07	10	35	3.46
25	20.06	8	31	3.42	22.42	10	34	3.45
26	19.83	8	29	3.33	22.44	10	34	3.49
27	20.07	11	29	2.63	20.76	10	30	2.74
28	20.10	8	29	3.44	22.10	7	33	3.50
29	20.17	10	31	3.17	21.15	10	30	3.19
30	20.10	10	29	2.86	21.61	10	30	3.06

Table S5. Descriptive statistics of daily mean temperatures from the 30 simulations in Paris, for the periods 1981–2010 and 2021–2050, corrected with the quantile mapping method.

	Paris (1981–2010)				Paris(2021–2050)			
**Simulation ID**	**Mean**	**Minimum**	**Maximum**	**Std. Dev**	**Mean**	**Minimum**	**Maximum**	**Std. Dev**
1	19.74	12	31	3.36	20.74	12	33	3.45
2	20.05	11	31	3.16	21.54	11	33	3.47
3	19.89	11	30	3.24	21.43	11	37	3.51
4	19.74	11	32	3.16	21.54	11	33	3.51
5	19.51	10	31	3.15	21.46	10	35	3.52
6	19.52	11	32	3.13	21.18	11	32	3.39
7	19.73	11	31	3.26	20.36	12	31	3.43
8	19.77	12	30	2.92	20.46	13	30	2.96
9	19.72	12	32	3.32	20.33	12	31	3.40
10	20.03	11	32	3.16	21.51	11	34	3.57
11	19.84	11	31	3.15	21.70	12	36	3.64
12	19.61	11	32	3.20	21.22	12	32	3.30
13	19.69	10	30	3.19	21.22	11	32	3.34
14	19.54	11	32	3.17	21.51	11	34	3.53
15	19.70	11	31	2.56	20.13	12	35	2.80
16	19.81	9	31	3.03	21.11	11	33	3.36
17	19.72	11	31	3.20	20.63	12	32	3.58
18	19.71	11	30	3.11	20.17	12	31	3.12
19	19.75	12	30	2.89	21.22	12	32	3.11
20	19.71	12	31	3.34	20.66	11	32	3.43
21	19.83	9	30	3.07	21.05	11	31	3.20
22	19.78	12	29	2.89	20.47	12	30	2.89
23	19.71	12	31	3.38	21.08	12	34	3.57
24	19.99	11	31	3.19	21.55	12	35	3.53
25	19.79	11	32	3.22	22.01	12	34	3.62
26	19.45	11	32	3.13	21.60	12	32	3.38
27	19.71	11	31	2.53	20.11	11	33	2.84
28	19.71	11	31	3.27	20.89	11	32	3.53
29	19.73	11	30	3.10	20.30	11	30	2.90
30	19.78	12	30	2.88	21.25	13	32	3.11

Table S6. R0R for each temperature unit in Montreal.

Temperature Unit	RR	95% CI
10	0.97	0.94, 1.01
11	0.99	0.94, 1.03
12	0.92	0.89, 0.95
13	0.94	0.92, 0.96
14	0.91	0.90, 0.93
15	0.92	0.91, 0.93
16	0.98	0.97, 1.00
17	0.97	0.97, 0.98
18	0.98	0.96, 1.00
19	0.95	0.95, 0.96
20	0.95	0.95, 0.96
21	0.97	0.96, 0.98
22	0.99	0.98, 0.99
23	1.02	1.01, 1.03
24	1.05	1.05, 1.06
25	1.10	1.09, 1.10
26	1.13	1.12, 1.14
27	1.18	1.17, 1.19
28	1.21	1.20, 1.22
29	1.24	1.21, 1.29

Table S7. RR for each temperature unit in Paris.

Temperature Unit	RR	95% CI
12	0.98	0.96, 1.01
13	0.97	0.95, 0.99
14	0.97	0.96, 0.99
15	0.99	0.98, 0.99
16	0.98	0.98. 1.00
17	0.97	0.96, 0.99
18	0.97	0.96, 0.98
19	0.97	0.96, 0.99
20	0.98	0.97, 1.01
21	0.99	0.97, 1.03
22	1.04	1.02, 1.06
23	1.06	1.03, 1.08
24	1.09	1.06, 1.11
25	1.12	1.08, 1.15
26	1.15	1.11, 1.19
27	1.18	1.14, 1.23
28	1.22	1.14, 1.27
29	1.23	1.13, 1.31

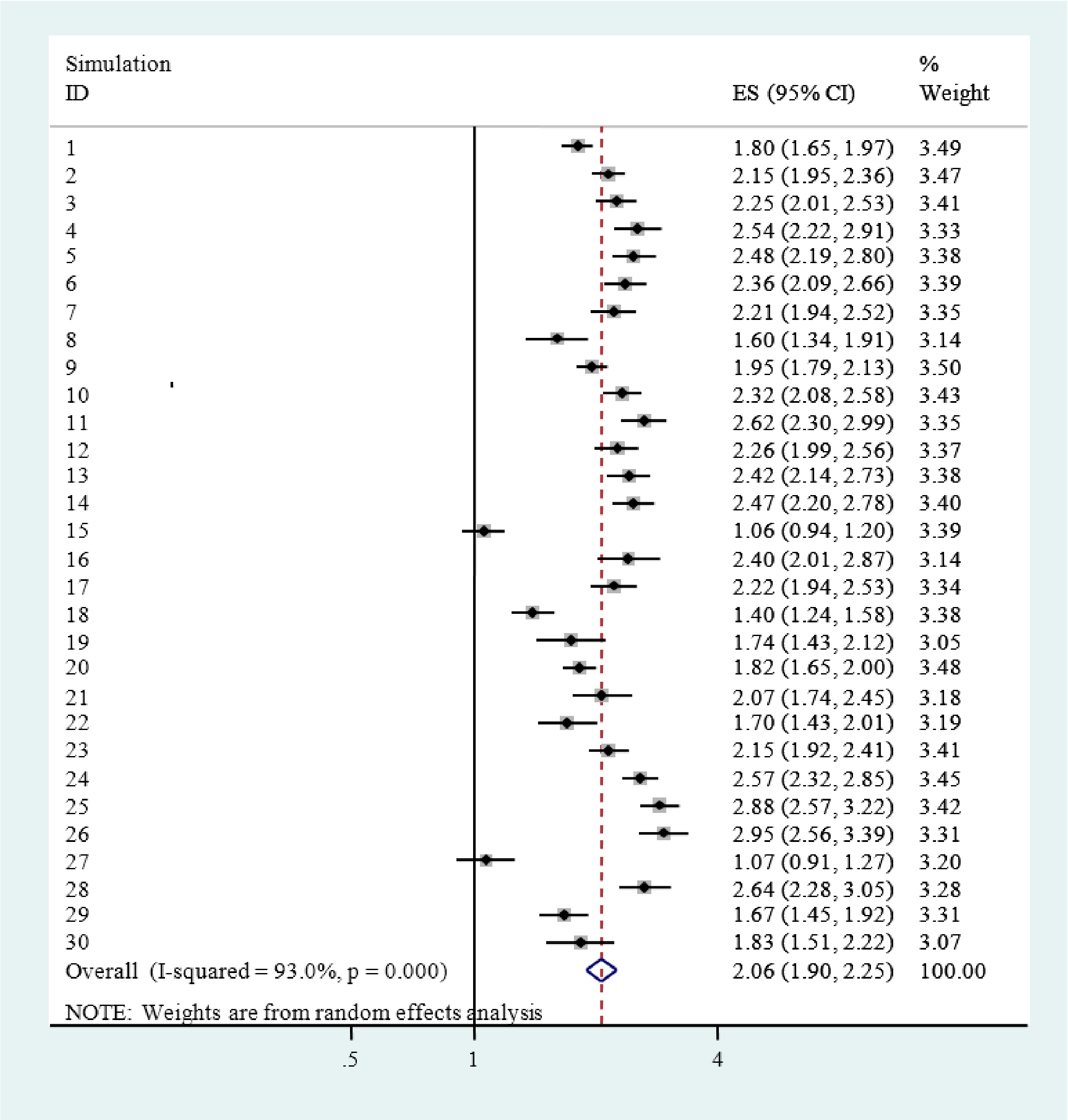

Figure S1. Meta-analysis of the impact of climate change on summer YLL disparities attributable to temperatures in Montreal; ES: Effect Size.

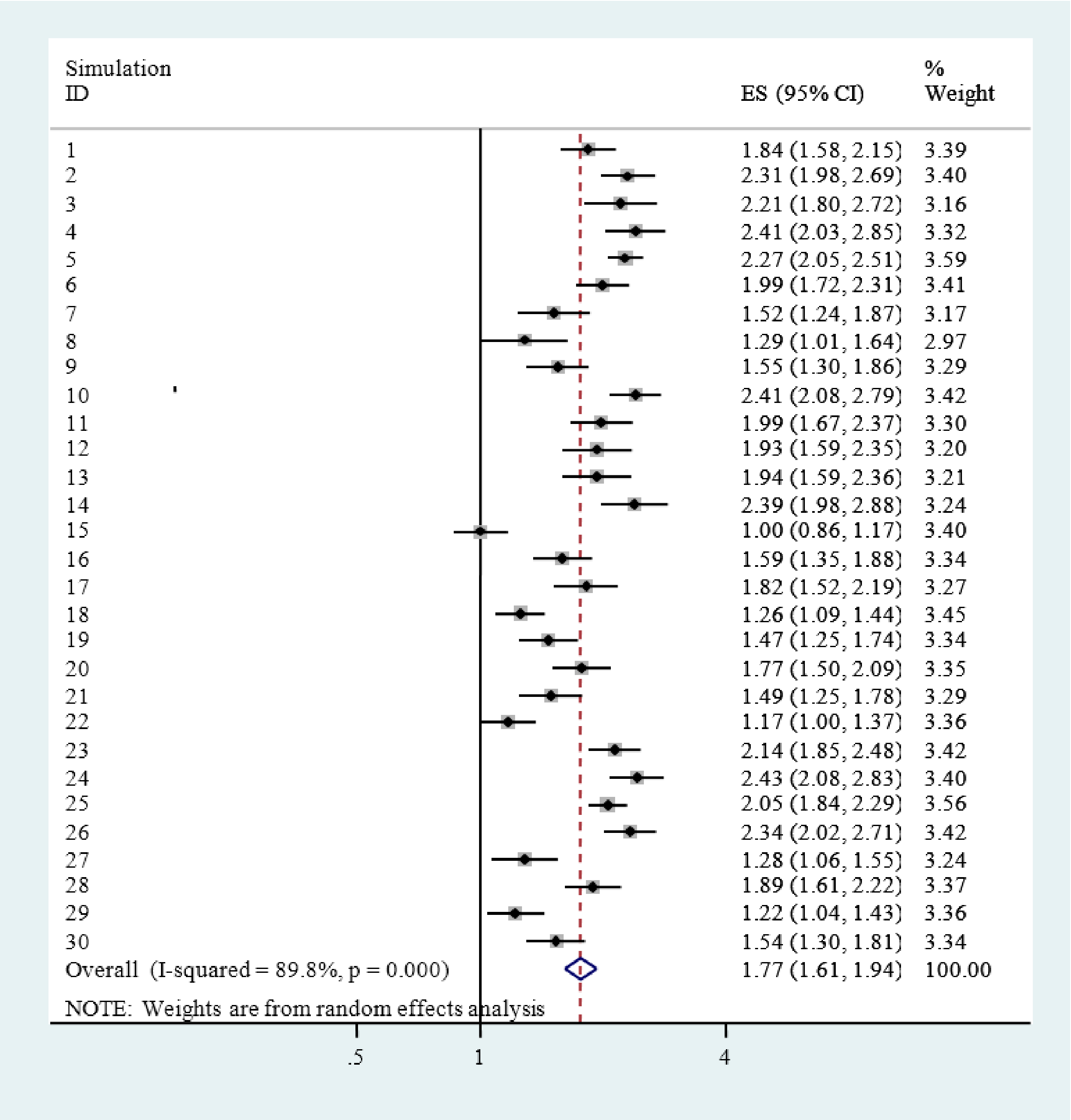

Figure S2. Meta-analysis of the impact of climate change on summer YLL disparities attributable to temperatures in Paris; ES: Effect Size.

Sensitivity Analyses

We conducted different sensitivity analyses related to our statistical analyses. We present and used RRs from regression analyses that did not adjust for relative humidity as it had minimal influence on the RRs (data not shown). Changing the degrees of freedom for secular trends had a minimal influence on RRs as well (data not shown).

We conducted different sensitivity analyses to make the comparison plausible between Montreal and Paris. These sensitivity analyses were related to the mortality data and the social vulnerability factor used. Because we did not have access to underlying cause of mortality, we only included Paris subjects older than 35 years old at the time of death to minimize this bias because accidental causes of death are

dominant in subjects under 35 years old. We also used this approach with the Montreal data. We used as social vulnerability indicator, a composite deprivation index for Paris [6] for a sensitivity analysis.

Conducting sensitivity analyses, our results remained unaltered by modifications of the population (using individuals >35 years of age for Montreal), and of the social indicators used (*i.e.*, composite deprivation index for Paris) (see the following Table 7S).

We estimated the impact of climate change on DYLLD by including changes in the population size in the future horizon of 2030. For Montreal, we used two demographic projections scenarios from the National Institute of Statistics (http://www.stat.gouv.qc.ca/statistiques/population-demographie/perspectives/population/popqcade_14.htm): one decreasing scenario (−6%) and one increasing scenario (+6%). For Paris, we used two demographic projections scenarios from the French INSEE (http://www.insee.fr/fr/themes/detail.asp?reg_id=99&ref_id=proj-dep-population): one decreasing scenario (−1%) and one increasing scenario (+1%). We calculated the ratio between future and historical newly produced rates of DYLDD.

Table S8. Sensitivity analyses results.

	Estimates	Using Individuals >35 years of Age *	Using a Composite Deprivation Index
**Montreal**	Mean daily estimates of DYLLD	363.21 years (SD = 333.62 years)	NA
	Summer rates of DYLLD attributable to temperature in historical period	36.11 years per 100,000 persons (95% CI: 16.56, 48.01)	NA
	Summer rates of DYLLD attributable to temperature in future period	23.56 years per 100,000 persons (95% CI: 15.56, 34.10) to 102.31 years per 100,000 persons (95% CI: 83.34, 123.18)	NA
	ICC overall Ratio	2.06 (95% CI: 1.92, 2.29)	NA
**Paris**	Mean daily estimates of DYLLD	NA	203.12 (SD = 206.69)
	Summer rates of DYLLD attributable to temperature in historical period	NA	15.13 years per 100,000 persons (95% CI: 8.63, 19.06)
	Summer rates of DYLLD attributable to temperature in future period	NA	11.52 years per 100,000 persons (95% CI: 7.85, 20.11) to 37.20 years per 100 000 persons (95% CI: 27.13, 44.31)
	ICC overall Ratio	NA	1.82 (95% CI: 1.66, 1.99)

* 8008 cases of death were excluded for the whole period (1990–2007); NA: Not Applicable.

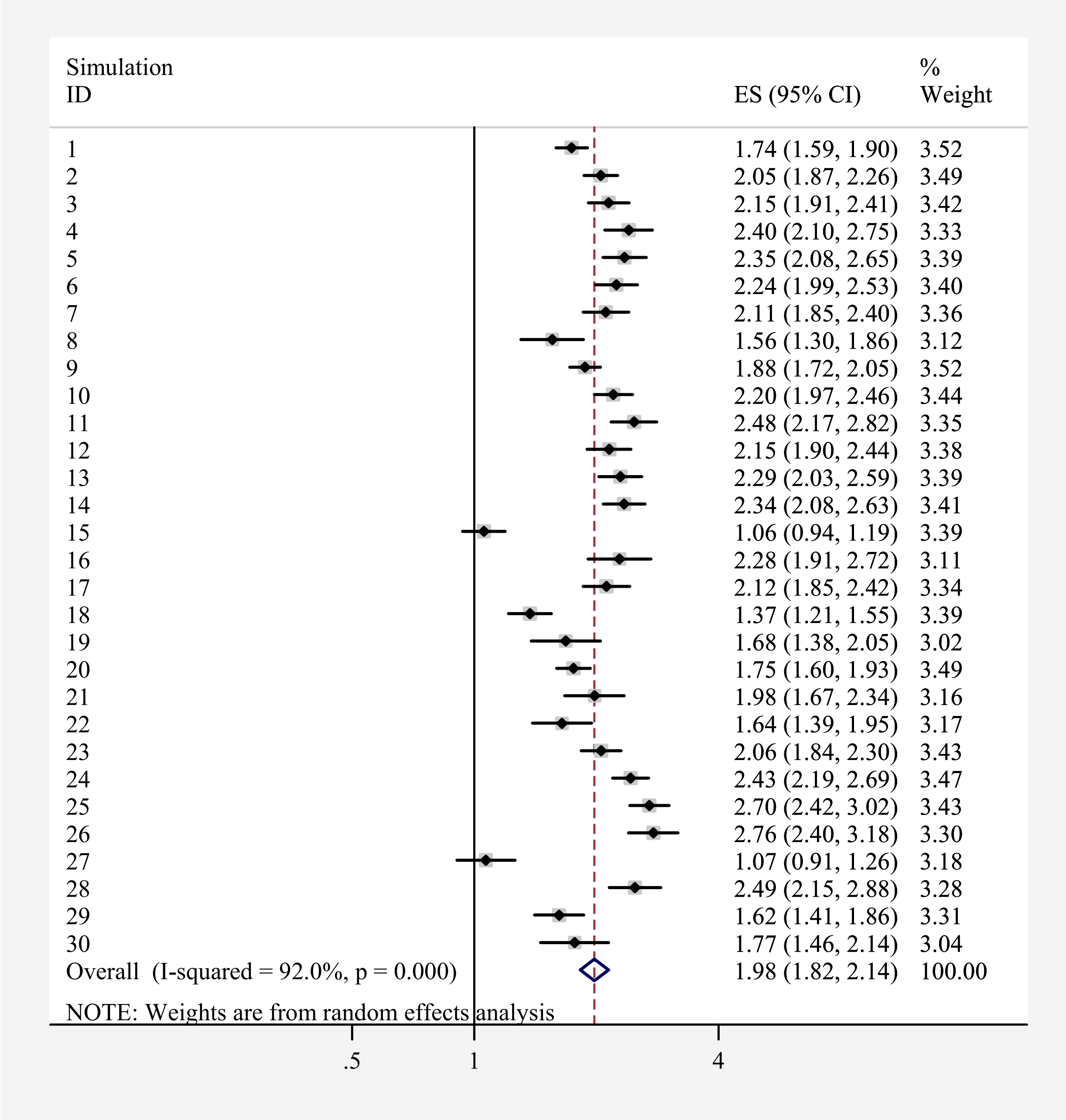

Figure S3. Meta-analysis of the impact of climate change on summer YLL disparities attributable to temperatures in Montreal with an increase scenario demographic projections *; ES: Effect Size. * http://www.stat.gouv.qc.ca/statistiques/population-demographie/perspectives/population/popqcade_14.htm.

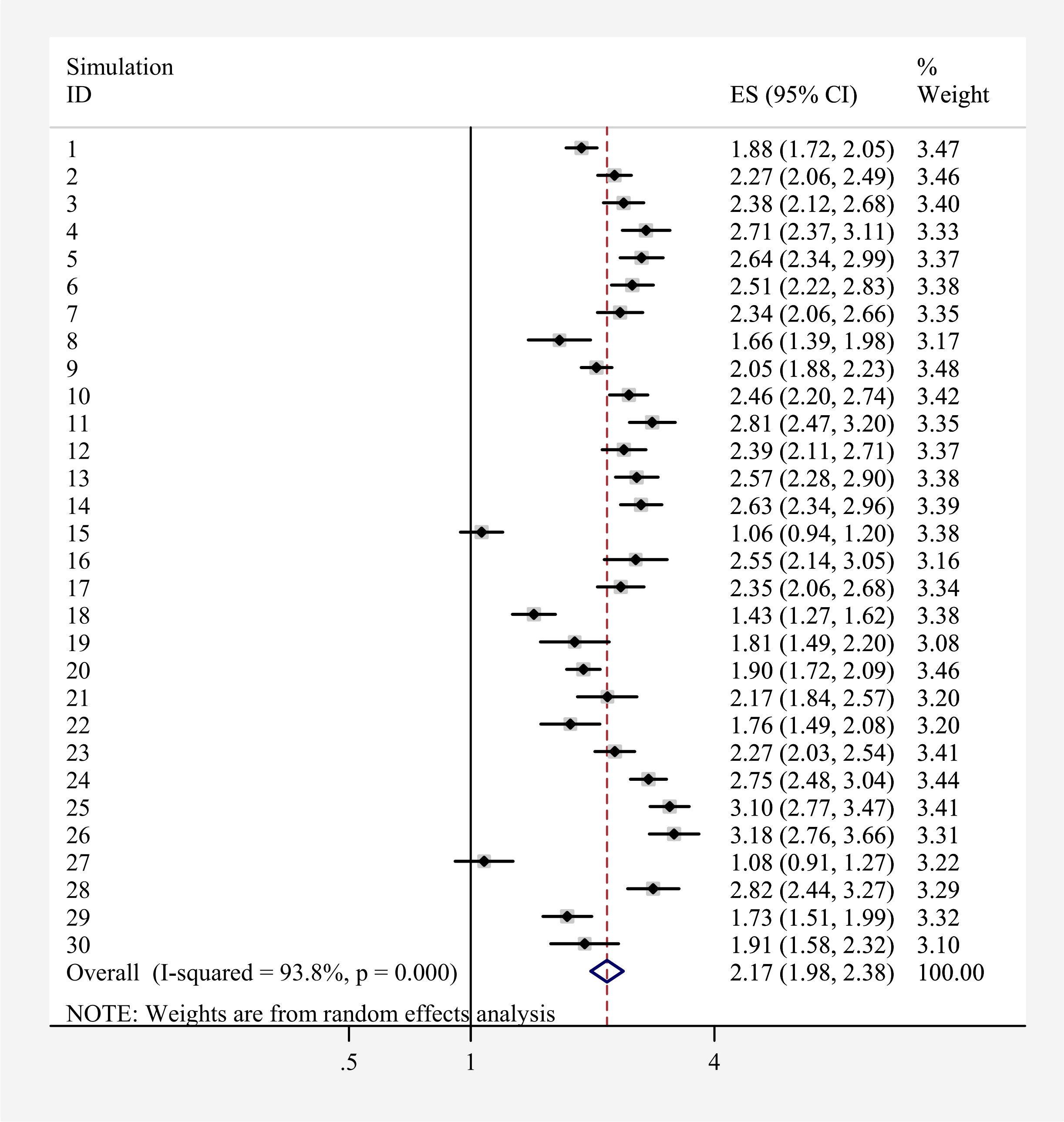

Figure S4. Meta-analysis of the impact of climate change on summer YLL disparities attributable to temperatures in Montreal with a decrease scenario demographic projections *; ES: Effect Size. ** http://www.stat.gouv.qc.ca/statistiques/population-demographie/perspectives/population/popqcade_14.htm.

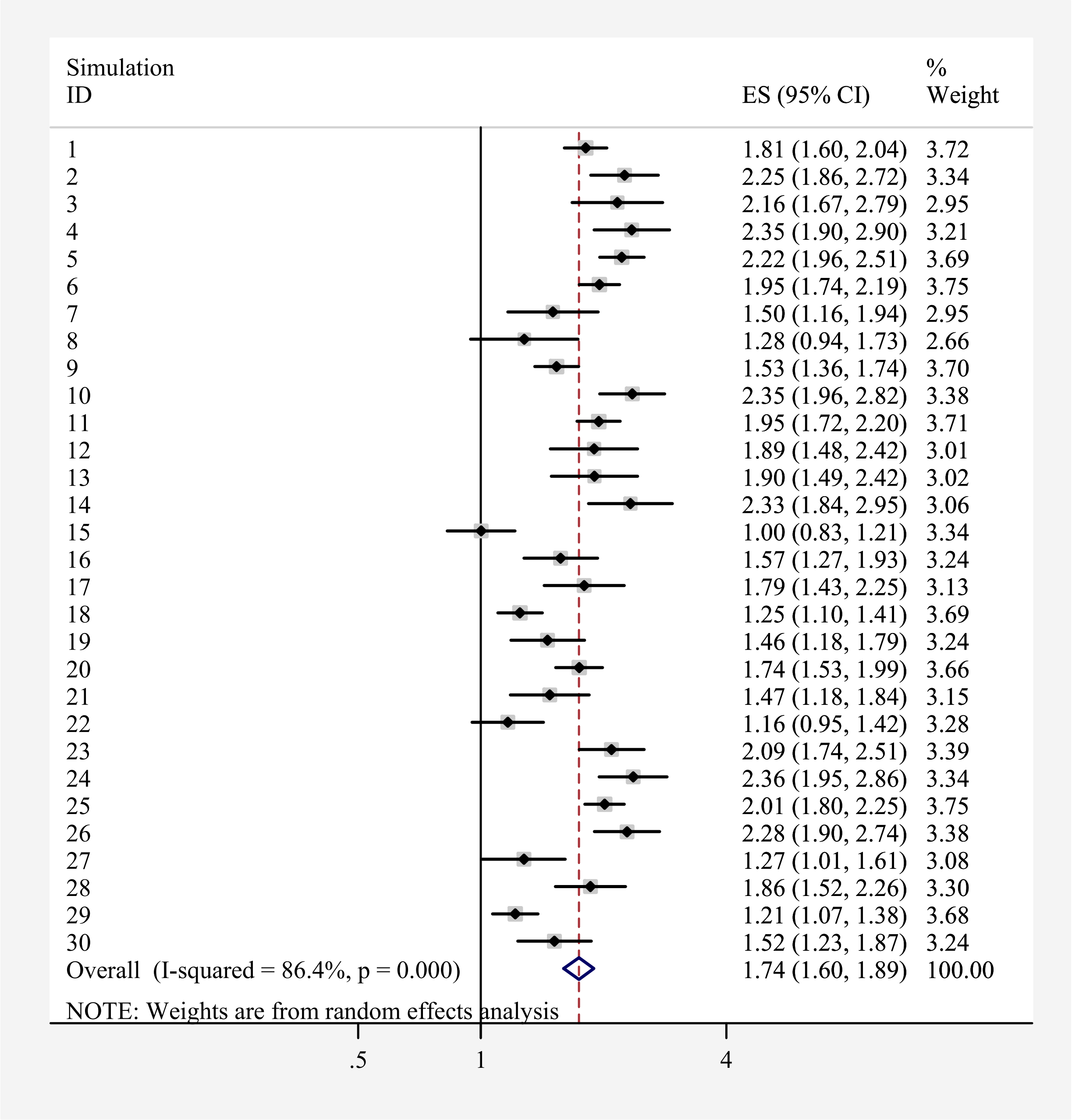

Figure S5. Meta-analysis of the impact of climate change on summer YLL disparities attributable to temperatures in Paris with an increase scenario demographic projections * ES: Effect Size. * http://www.insee.fr/fr/themes/detail.asp?reg_id=99&ref_id=proj-dep-population.

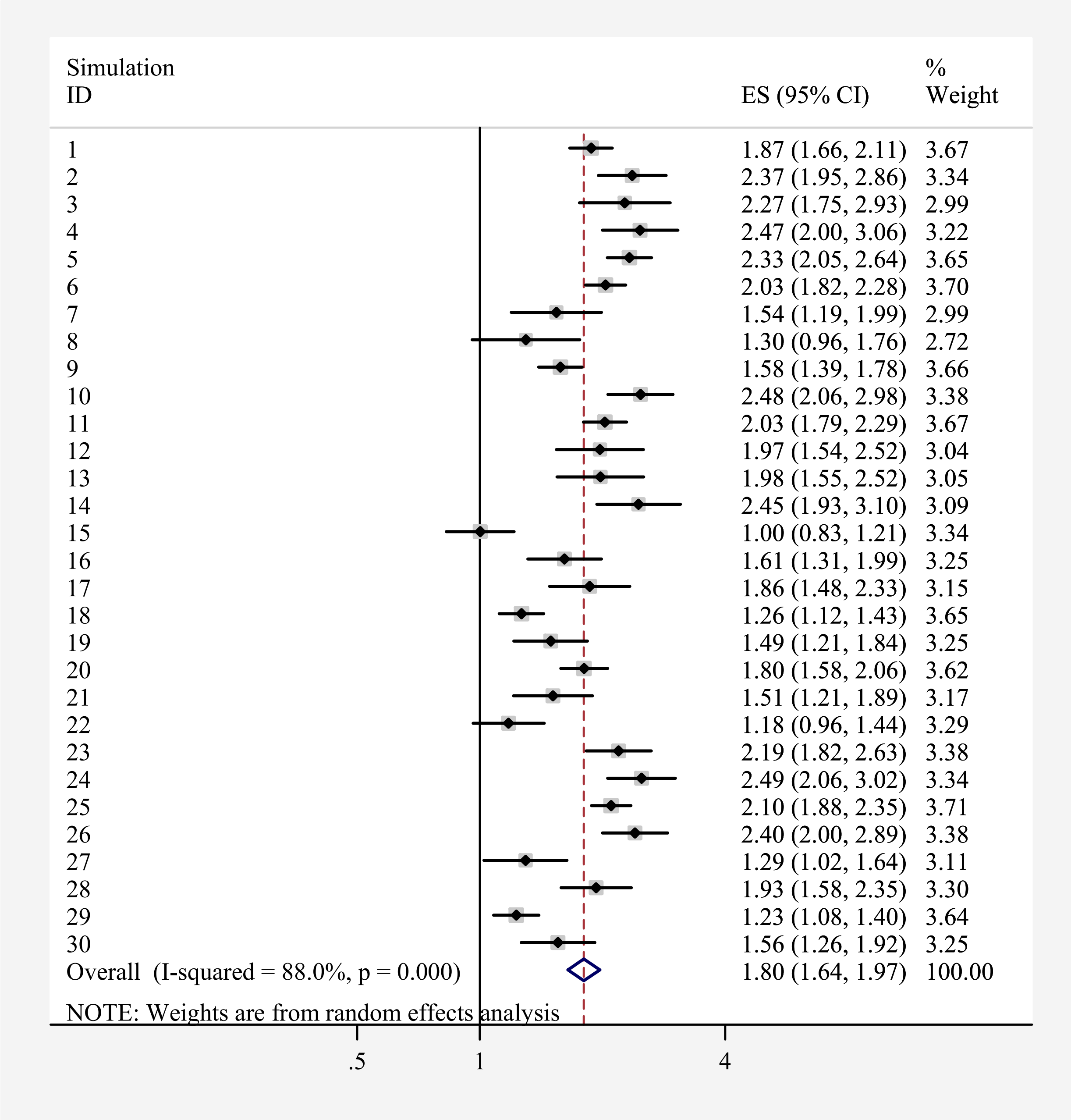

Figure S6. Meta-analysis of the impact of climate change on summer YLL disparities attributable to temperatures in Paris with a decrease scenario demographic projections * ES: Effect Size. ** http://www.insee.fr/fr/themes/detail.asp?reg_id=99&ref_id=proj-dep-population.

References

1. Taylor, K.E.; Stouffer, R.J.; Meehl, G.A. An overview of CMIP5 and the experiment design. *Bull. Am. Meteorol. Soc.* **2012**, *93*, 485–498.
2. Themeßl, M.J.; Gobiet, A.; Heinrich, G. Empirical-statistical downscaling and error correction of regional climate models and its impact on the climate change signal. *Clim. Change* **2012**, *112*, 449–468.

3. Bhaskaran, K.; Gasparrini, A.; Hajat, S.; Smeeth, L.; Armstrong, B. Time series regression studies in environmental epidemiology. *Int. J. Epidemiol.* **2013**, *42*, 1187–1195.
4. Institut Statistiques Québec. 2001. Available online: http://www.stat.gouv.qc.ca (accessed on 12 March 2014).
5. INSEE. 2006. Available online: www.insee.fr (accessed on 12 March 2014).
6. Lalloué, B.; Monnez, J.M.; Padilla, C.; Kihal, W.; Le Meur, N.; Zmirou-Navier, D.; Deguen, S. A statistical procedure to create a neighborhood socioeconomic index for health inequalities analysis. *Int. J. Equity Health* **2013**, doi:10.1186/1475–9276–12–21

© 2015 by the authors; licensee MDPI, Basel, Switzerland. This article is an open access article distributed under the terms and conditions of the Creative Commons Attribution license (http://creativecommons.org/licenses/by/4.0/).
